# Supplementary material for: Complex‐centric proteome profiling by SEC‐SWATH‐MS
Source: Mol Syst Biol. 2019 Jan 14;15(1):e8438. doi: 10.15252/msb.20188438 (PMC6346213; doi:10.15252/msb.20188438)
Supplement: Supplementary file 7 — Dataset EV6 [file MSB-15-e8438-s007.zip › feature_plots_bioplex/Q86Y82.pdf]

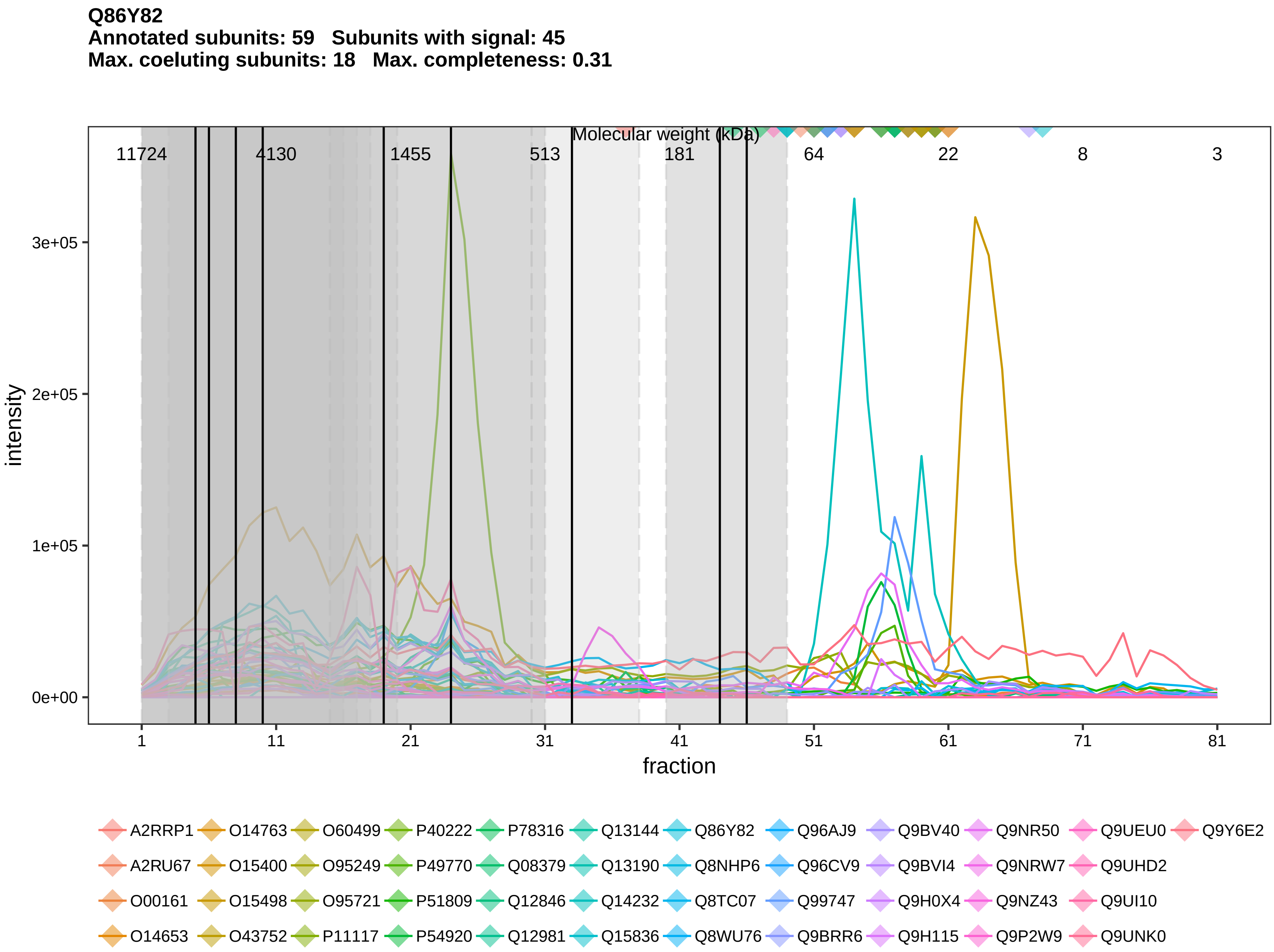
Q86Y82
Annotated subunits: 59 Subunits with signal: 45
Max. coeluting subunits: 18 Max. completeness: 0.31
Molecular weight (kDa)
11724
4130
1455
513
181
64
22
8
3
3e+05
2e+05
1e+05
0e+00
intensity
1
11
21
31
41
51
61
71
81
fraction
A2RRP1
O14763
O60499
P40222
P78316
Q13144
Q86Y82
Q96AJ9
Q9BV40
Q9NR50
Q9UEU0
Q9Y6E2
A2RU67
O15400
O95249
P49770
Q08379
Q13190
Q8NHP6
Q96CV9
Q9BVI4
Q9NRW7
Q9UHD2
O00161
O15498
O95721
P51809
Q12846
Q14232
Q8TC07
Q99747
Q9H0X4
Q9NZ43
Q9UI10
O14653
O43752
P11117
P54920
Q12981
Q15836
Q8WU76
Q9BRR6
Q9H115
Q9P2W9
Q9UNK0
